# Understanding the role of KABAM in siddha medicine: A scientific analysis of dietary and lifestyle factors

**DOI:** 10.6026/973206300210930

**Published:** 2025-05-31

**Authors:** Sri Sakthi Logisha M., Vinodini Ramamoorthy, Nivetha G., Muthukumar N.J., Mahalakshmi V.

**Affiliations:** 1 Department of Sirappu Maruthuvam, National Institute of Siddha, The TamilNadu Dr MGR Medical University, Chennai, India; 2 Department of Clinical Research, Research Associate (Siddha), Central Council Research in Siddha, Ministry of Ayush, Government of India, Kuyavarpalayam, Puducherry - 605013, India; 3 Department of Varmam Maruthuvam, National Institute of Siddha, The TamilNadu Dr MGR Medical University, Chennai, India; 4 Diretor General, Central Council for Research in Siddha, Chennai, India; 5 Department of Siddhar Yoga Maruthuvam, National Institute of Siddha, The TamilNadu Dr MGR Medical University, Chennai, India

**Keywords:** KABAM, THEGA ELAKKANAM, body constitution, diet

## Abstract

KABAM is one of the three humors (connected to humorism, the humoral theory, or humoralism) in the Siddha system of medicine which is a combination of the earth and water. Several classical siddha literatures stated the importance of the tri-humoral personalities of individuals. Hence, analysis of body constituent (in Tamil language "THEGA ELAKKANAM") has been in practice in alternative systems of medicine. Related guidelines are available for identifying specific body constitution types, dietary recommendations and disease susceptibility. Therefore, it is of interest to discuss the "THEGA ELAKKANAM" characters and related diet linked with KABAM.

## Background:

The Siddha system is one of the oldest systems of medicine in India. Eighteen SIDDHAR's were said to have contributed to the development of this medical system [1]. The diagnosis in the Siddha system of medicine involves identifying its cause. Identification of causative factors is through the unique diagnostic tool, namely ENVAGAI THERVU (eight types of test), which includes the examination of pulse, tongue and the complexion of the body, nature of voice, eyes, stool and urine, perception by palpation and the status of the digestive system [2]. It emphasizes that disease management is oriented not merely to disease but also to consider the patient's body texture, environment, meteorological consideration, age, sex, race, habits, mental status, habitat, diet, appetite, physical condition and psychological constitution, etc. Thus the treatment has to be individualistic, which ensures better diagnosis and treatment [3]. Siddha system is evolved based on ninety-six tools called 96 THATHUVAM (theory), which include physical, physiological, psychological and intellectual aspects of every human being. Three vital life factors are responsible for good health among the ninety-six tools. They are formed by the appropriate combination of elements from intra-uterine life. VATHAM/VALI is made by the mixture of the elements air and space; the element fire creates PITHAM/AZHAL and KABAM/IYAM is formed by the combination of the element earth and water [4]. The cornerstone of Siddha medicine for healthy living is food and lifestyle. "Food itself is medicine and medicine itself is food" is one of the basic principles of the Siddha system of medicine [5]. Food selection according to the landscape, place and lifestyle leads to a healthy life. Therefore, it is of interest to discuss the "THEGA ELAKKANAM' characters and diet for KABAM.

## Bio-energy water (IYAM/ KABAM):

One of the three humours of the functional constitution of the body represents the elements water and earth. KABAM is the principle of stabilizing energy and governs growth in the body and mind and is concerned with structure, stability, lubrication and fluid balance. It dominates the head and neck region and exhibits itself in five forms, *i.e.*, AVALAMBAGAM, KILAETHAM, POTHAGAM, THARPAGAM and SANTHIGAM and is eliminated from the body through urine [3, 5].

## Serum (AVALAMBAGAM - ALI IYAM):

It lies in the lungs and helps in respiration. It causes firmness of the limbs. It is vital among all types of KABAM. It controls the other KABAM types and maintains equilibrium [6, 7].

## Saliva (KILAETHAGAM - NEERPIIYAM):

It lies in the stomach; it mixes the consumed food and water and promotes the digestive process [5, 8].

## Lymph (POTHAGAM - SUVAIKAANIYAM):

It lies in the tongue and helps to realize the taste of consuming foods [5, 8].

## Cerebrospinal fluid (THARPAGAM - NIRAIVUIYAM):

Sustaining in the head gives a refrigerant effect to cool the eyes and other sense organs [5, 8].

## Synovial fluid (SANTHIGAM - ONRIIYAM):

Sustaining the joints makes them move freely and easily [7, 8].

## Anatomical and physiological sites of KABAM:

The primary locations where KABAM resides include SAMANA VAYU (type of VALI), SUZHUMUNAI, semen, head, AKKINAI (One of the 6 energy station - resides in between eyebrows), tongue, uvula, fat, bone marrow, blood, nose, chest, nerves, bones, brain, large intestine, eyes and joints. These sites are where KABAM predominantly functions [5].

## Natural characteristics of KABAM:

The natural characteristics include stability in its natural state, lubrication, the structural integrity of joints and tolerance. This tolerance extends to hunger, thirst, sorrow, anxiety and heat [5].

## Body constituents (THEGA ELAKKANAM):

## IYAM/ KABAM THEGI:

Persons with KABAM constitution will have a heavy, sweaty body, a strong will, a reddish complexion, infatuation, a thundering voice, humility, untruthful speech, a voracious appetite, a preference for sweet food items, a cough and lethargy [10]. The characteristics of KABAM constituent are described below;

[1] Body frame: They have broad, heavy bones and a heavy bodily structure, which is certainly indicative of their ability to store energy. They have noticeably short and squarish feet and toes [6, 7].

[2] Weight: They have a natural tendency to gain a lot of weight due to the inherent character of energy conservation and aversion to energy spending. As a result, they may acquire weight easily but find it difficult to lose it [7].

[3] Walk: They walk slowly and steadily, relaxed manner, with no accompanying movements [8].

[4] Teeth: They have huge, dazzling teeth that rarely have to be maintained [9].

[5] Digestive power: They have weak digestive systems and are more prone to digestive disorders [10].

[6] Ability to bear hunger: They have an excellent tolerance for hunger and can last for long durations without eating [5].

[7] Thirst: They rarely experience thirst [5, 6].

[8] Quantity of food: They have a normal appetite and eat an appropriate quantity of food [7, 8].

[9] Groups of desired tastes: They prefer astringent and bitter-tasting foods [8].

[10] Foods desired: They like warm and dry food [7, 8].

[11] Bowel movements: They have regular stools that are excreted slowly [10, 11].

[12] Perspiration: They perspire normally in any climate [6, 15].

[13] Sleep: They have intense and excessive sleep patterns because they naturally prioritize energy conservation [6].

[14] Dreams: They frequently dream about water, clouds and romance [8].

[15] Personality traits: They value peacefulness and pleasure in their homes and families. They are serious, stable and quiet people with a lot of patience, courage and humility, which can lead to laziness, attachment, possessiveness and avarice in excess [7, 10]. They are naturally compassionate and their solid maternal instincts arise from the earth element's domination in them [13].

[16] Speech and voice qualities: They speak slowly without any enthusiasm. Their voices are sweet, clear and resonant and their pitch is substantially lower than that of VATHAM OR PITHAM people. They will only engage in conversation if they have something significant to say and it is a pleasure to listen to them [11].

[17] Energy spending: They exhibit a steady flow of energy [14].

[18] Performances of activities: They work very slowly and consistently. Their pace is calm and gradual, undisturbed by anything [7, 12].

[19] Excitability: They respond slowly to the excitement, showing no signs of understanding the impact of anything immediately. They take their time, which results in a relatively calm response showing their unwillingness to spend energy quickly [7].

[20] Grasping power: They are slower at learning or absorbing new concepts [12].

[21] Memory: They have an extraordinarily long memory and don't forget anything easily, yet they need to be told anything multiple times before it fully registers. Their innate capacity for storage helps the mind retain every detail for an extended period [8].

[22] Nature of moods: They are significantly more relaxed and reliable but exhibit unusual behavior symptoms [7, 15].

[23] Attitude to problems or difficulties, their characteristics emotions: They are peaceful, slow and steady when confronted with difficulties and challenges since they naturally avoid conflicts. While dealing, they have a constant resolution, but this can occasionally manifest as passivity or inactivity [8-12].

## IYAAZAL/ KABA PITHA THEGI:

KABAPITHA people have a rosy/ox gall/greenish-black complexion that is tinged somewhat green and often have reddish body hair. These people have oily, sensitive skin and are capable of arousing and igniting sexual desire. They also have outstanding verbal skills and a clear tone of voice or an explosive voice. These individuals are primarily driven toward the path of wisdom, committed to the way of truth and crave sour, sweet and spicy foods. Under the influence of the KABA nature's solidification, their PITHA nature's sharp intelligence is strongly channeled [3, 10].

## IYAVALI/KABA VATHA THEGI:

KABAVATHAM persons have a strong body, a dark/blackish-red or rosy complexion and a strong sexual urge. They intend to acquire power, obtain access to supernatural forces and develop a passionate interest in research. They exhibit a particular love of learning, adoration for the elderly, infatuation, boldness and scriptural scholarship. They naturally possess the capacity to take the initiative and move things ahead. They primarily crave sour and pungent foods. In this type of constitution, the cooling tendency of both KABAM and VATHAM leads to circulatory problems. In terms of the positive, the grounding quality of the KABA force combined with the creative traits of VATHAM makes leading personalities, rulers of a nation or corporate sectors and/or in areas of new gadgets and mechanisms invention [3, 7].

## Characteristics of deranged KABAM:

KABAM imbalance in Siddha medicine is characterized by various physiological and pathological changes that reflect a disturbance in its natural qualities. The qualities of balanced and imbalanced KABAM are described in Table 1. However, an imbalance in KABAM leads to various physical and mental disturbances. Physically, it manifests as sluggishness, fatigue and heaviness, often slowing down metabolism and digestion. Excess KABAM increases mucous production, leading to respiratory conditions like asthma and bronchitis, along with symptoms such as hyper salivation, chills, or pale skin. Psychologically, an imbalanced KABAM can cause emotional detachment, possessiveness, or stubbornness, makes individuals less adaptable and more rigid in their thoughts or actions. Overdependence and excessive attachment are also common [3, 5]. Factors like diet, lifestyle, seasonal changes and stress influence the balance of KABAM. The characteristics of deranged KABAM, as detailed in various Siddha texts are outlined in Table 2.

## Diet for IYAM/ KABAM diseases:

The dietary recommendations for managing KABAM (IYAM) diseases, as outlined in the Siddha literature PATARTTAKUNACINTAMANI, focus on food items like Greater galangal (Alpinia galangal), Radish (Raphanus sativus), Brinjal (Solanum trilobatum), Bishop's weed (Carum copticum), Seeds of holy basil (ocimum sanctum), Wood apple (Feronia Elaphantum), Cow's milk, Honey, Burnt ash of Peacock's feather that help to balance excess heat and phlegm in the body. The general dietary advices for IYAM or KABAM constituent are outlined in Table 3.

## Discussion:

KABAM (IYAM) is a fundamental humor in Siddha medicine, playing a crucial role in maintaining body structure, fluid balance and immunity. It governs essential physiological functions, including respiration, digestion and the regulation of cerebrospinal fluid. When in balance, KABAM ensures strength, endurance and emotional stability. However, an imbalance can lead to various disorders, including respiratory, metabolic and psychological conditions [1]. Physiologically, KABAM maintains tissue integrity, regulates fluid balance, supports the lymphatic system, detoxifies the body and enhances immunity. A balanced state of KABAM helps prevent congestion, inflammation and digestive disturbances. Conversely, excess KABAM leads to weight gain, sluggish digestion and respiratory issues such as sinusitis, asthma and arthritis [3]. Individuals with a KABAM-dominant constitution typically have sturdy bodies and slow metabolisms. They are often calm, patient and grounded but may experience lethargy, mental stagnation and emotional attachment when imbalanced [5]. Diet plays a significant role in managing KABAM imbalances. The Siddha tradition emphasizes the concept that "food itself is medicine and medicine itself is food," highlighting the significance of diet in health maintenance and disease prevention. Foods that are heavy, cool and greasy aggravate KABAM, whereas warm, dry and light foods help neutralize excess moisture. Spicy and bitter foods such as dry ginger, mustard and pepper aid digestion, while avoiding dairy, fried foods and sugary items helps prevent dampness. Regular exercise, including walking or yoga, promotes balance by mobilizing excess water and preventing weight gain. Siddha medicine suggests therapeutic interventions for KABAM imbalances, including detoxification therapies like purgation and sweating, as well as internal medicines. When KABAM is disturbed, it can also affect mental health, leading to sluggishness and depression that should be restored by mindfulness, meditation and physical activity. Maintaining balance in KABAM is essential for overall health. Proper diet, regular exercise and appropriate therapeutic interventions play a vital role in preventing diseases and promoting physical and mental well-being. Siddha medicine emphasizes the importance of harmonizing KABAM to achieve holistic health.

## Conclusion:

The concept of KABAM (water and earth features) in siddha medicine is panoramic because it indicates the need for balance to physical, mental and emotional well-being. Hence, tailored dietary and lifestyle habits help reverse imbalances and promote health on all levels using awareness of different physio-psychological characteristics of KABAM. Thus, siddha medicine is holistic in nature with a purpose and helps reach a state of balance with vitality using awareness of KABAM features.

## Ethical approval:

The authors declare that due to a literature review this study does not need any ethical approval.

## Consent to participate:

Not applicable

## Author contributions:

All authors have equally contributed in the manuscript preparation like concept, designing and collection of the data, as well as the writing and revision of the article.

## Funding:

The authors declare that no funds, grants, or other support were received during the preparation of this manuscript.

## Competing interests:

The authors have no relevant financial or non-financial interests to disclose.

## Availability of data and materials:

All the data's are available with authors upon reasonable request

## Source of funding:

No funds, grants, or other support was received for this work.

## Figures and Tables

**Table 1:** Qualities expressed by IYAM/ KABAMTHODAM when in balance and imbalance

**KABAM [12].**	
Balance	Imbalance
Steady	Sluggish
Loving	Possessive
Peaceful	Insensitive
Caring	Attached
Consistent	Stubborn/rigid

**Table 2:** Characters of deranged IYAM/ KABAMKUTRAM IN siddha literature

	**Symptoms**	**References**
1	The deranged KABAM causes lethargy, cold, chillness, excessive mucous secretion and gradually it weakens the body's natural strength and vitality.	[14]
2	The skin appears pale, cough; vomiting, fainting, excessive salivation, chest discomfort and difficulty in breathing become prominent.	
1	Tongue become pale and taste like sweet	[16]
2	Sudden difficulty in walking	
3	Increased urination, body become pale like cotton	
4	Sudden loss of physical strength and become weak to do any work	
1	Body become dry, withered, pale, chills and shivers	[17]
2	Unable to take food	
3	Hiccough, cough, exhalation, stunning, sweating,	
4	Discomfort in Chest and ribcage	
5	Bleeding	
6	Smoothness and sweet taste in tongue	
7	Hyper salivation	
8	Painful and burning urination	
1	Hyper salivation	[5, 18]
2	Indigestion	
3	Heaviness of body	
4	Reduction in physical strength	
5	Chillness and paleness of body	
6	Muscle flaccidity	
7	Cough, Wheeze, Asthma, Excessive sleep	
1	A sensation of the body being squeezed or compressed is quickly felt	[6]
2	Itching and feels cold of the body	
3	Lower limb swelling and pallor of face	
4	Giddiness	
5	Reduction in appetite and Loss of appetite.	
6	Increased sleep	
7	Sweet taste in mouth	
1	Runny nose	[19]
2	Chest congestion due to phlegm	
3	Hoarseness of voice and cough	
4	Stomach cramps with white, cold and loose stools	
1	Sweating	[20]
2	Increased salivation and Smooth tongue	
3	Urine and stool become whitish in colour	
4	Heaviness in the body	
1	Sweating followed by chills	[21]
2	Excessive salivation in the mouth	
3	Sweet taste and smoothness on the tongue	
4	Paleness of body	
1	Weight loss	[22]
2	Cough	
3	Bilateral rib pain, Fatigue	
4	Hematemesis	
	Due to increased IYAM, heat may rise at any time, causing a feeling of heat and an increase in thirst	[11]
	The rise in internal heat stimulates the brain, leading to symptoms such as bleeding, swelling and phlegm build-up in the chest, which causes coughing.	[17]
	This imbalance also results in fatigue and tuberculosis	

**Table 3:** Dietary advice for IYAMTHEGI (body constitution)

	**Tamil name / Common name**	**Botanical / Scientific name**
Grains	Raw rice	Oryza Sativa
	Boiled rice	Oryza Sativa
	Millets	-
	Corn	Zea mays
	Barley	Hordeum vulgare
Cereals	Green gram	Vigna radiate
	Red gram	Cajanus Cajan
	Horse gram	Macrotyloma Uniflorum
	Chickpea	Cicer arietinum
	Peanut	Arachis Hypogaea
	Bengal gram	Cicer arietinum
	Pigeon pea	Cajanus Cajan
	Sesame	Sesamum Indica
	Flax seeds	Linum Usitatissimum
Vegetables	Drumstick	Moringa Oleifera
	Bottle gourd	Lageneria Siceraria
	Snake gourd	Trichosanthes Cucumerina
	Bitter gourd	Momordica Charantia
	Carrot	Daucus Carota Sativus
	Cucumber	Cucumis Sativus
	Tomato	Solanum Lycopersicum
	Cauliflower	Brassica oleracea botrytis
	Garlic	Allium sativum
	Lettuce	Lactuca Sativa
	Celery	Apium Graveolens
	Broccoli	Brassica oleracea italic
	Horseradish	Armoracia rusticana
	Broad bean	Lablab purpureus
	Yellow fruit night-shade	Solanum Surattense
	Ridge gourd	Luffa acutangula
	Elephant yam	Amorphophallus Paeoniifolius
	Jack fruit	Artocarpus Heterophyllus
	Banana stem	Musa paradisiaca
	Radish	Raphanus Sativus
	Pumpkin	Cucurbita pepo
Greens	Ivy Gourd	Coccinia Grandis
	False amaranth leaves	Digera Muricata
	Puncture vine	Tribulus Terrestris
	Spleen amaranth leaves	Marsilea Quadrifolia
	Creeping wood sorrel	Oxalis corniculata
	Indian acalypha leaves	Acalypha Indica
	India pennywort	Centella Asaitica
	Sessile joyweed leaves	Alternanthera Sessilis
	Madras pea pumpkin leaves	Mukia Maderaspatana
	Drumstick leaves	Moringa Oleifera
	Chocolate weed leaves	Melochia Corchorifolia
	Flamingo Feather	Celosia arhentea
	Sand herbage	Lactuca Sativa
	Amaranth leaves	Amaranthus Polygonoidus
	MULLIKEERAI	Amaranthuss Pinosus
	MUSUTTAI	Rivea ornate
	Red Sorrel leaves	Hibiscus cannabinus
	Black night shade	Solanum nigrum
	Spinach	Portulacaquadrifida
	Indian aloe	Aloe vera
Spices	Dry ginger	Zingiberofficinale
	Cardamom	Elettariacardamomum
	Pepper	Piper nigrum
	Red chilli	Capsicum annuum
	Long pepper	Piper longum
	Mustard	Brassica juncea
	Coriander	Coriandrum Sativum
	Fenugreek	Trigonella Foenum-graecum
	Cumin	Cuminum cyminum
	Clove	Syzygium aromaticum
	Asafoetida	Ferula asafoetida
	Cinnamon	Cinnamomum verum
Fruits	Mango ripe	Mangifera Indica
	Gooseberry	Ribesuva-crispa
	Lemon	Citrus limon
	Pomegranate	Punica Granatum
	Pineapple	Ananas Comosus
	Grapes	Vitis Vinifera
	Cherries	Prunus Avium
	Papaya	Carica papaya
	Strawberry	Fragariaananassa
	Goji berries	Lyciumbarbarum
	Sugarcane	Saccharumofficinarum
	Wood apple	Limonia Acidissima
Dry fruits	Cashew	Anacardium Occidentale
	Dry figs	Ficus racemose
	Raisins	Vitis Vinifera
Oils	Sesame	Sesamum indicum
	Mustard	Brassica juncea
	Sunflower	Helianthus annuus
Milk and Milk products	Cow's milk	-
Non-vegetarian foods	Mutton	Ovisaries
	Chicken	Gallus gallusdomesticus
	Honey	-
Salted and dried vegetable (Vatral)	Turkey berry	Solanum Torvum
	Black night shade	Solanum nigrum
	Yellow-berried nightshade	Solanum xanthocarpum
	Snake guard	Trichosanthes Cucumerina
	Broad bean	Lablab purpureus
	Brinjal	Solanum Trilobatum
	Tender Indian gooseberry	Phyllanthus Emblica
	Throny caper	Capparis Horrida
	Tender Citron fruit	Citrus medica
Pickles	Pepper	Piper nigrum
	Tender turmeric	Curcuma longa
	Turkey berry	Solanum Torvum
	Tender mango	Mangifera Indica
	Bananastem	Musa paradisiaca
	Tender Fig	Ficus racemose
	Tender Bitter gourd	Momordica Charantia
	Yellow-berried nightshade	Solanam Xanthocarpum
